# Comodulation Enhances Signal Detection via Priming of Auditory Cortical Circuits

**DOI:** 10.1523/JNEUROSCI.0656-16.2016

**Published:** 2016-12-07

**Authors:** Joseph Sollini, Paul Chadderton

**Affiliations:** Department of Bioengineering, Imperial College London, London SW7 2AZ, United Kingdom

**Keywords:** broadband, coherence, masking release, noise, opsin, pyramidal cell

## Abstract

Acoustic environments are composed of complex overlapping sounds that the auditory system is required to segregate into discrete perceptual objects. The functions of distinct auditory processing stations in this challenging task are poorly understood. Here we show a direct role for mouse auditory cortex in detection and segregation of acoustic information. We measured the sensitivity of auditory cortical neurons to brief tones embedded in masking noise. By altering spectrotemporal characteristics of the masker, we reveal that sensitivity to pure tone stimuli is strongly enhanced in coherently modulated broadband noise, corresponding to the psychoacoustic phenomenon comodulation masking release. Improvements in detection were largest following priming periods of noise alone, indicating that cortical segregation is enhanced over time. Transient opsin-mediated silencing of auditory cortex during the priming period almost completely abolished these improvements, suggesting that cortical processing may play a direct and significant role in detection of quiet sounds in noisy environments.

**SIGNIFICANCE STATEMENT** Auditory systems are adept at detecting and segregating competing sound sources, but there is little direct evidence of how this process occurs in the mammalian auditory pathway. We demonstrate that coherent broadband noise enhances signal representation in auditory cortex, and that prolonged exposure to noise is necessary to produce this enhancement. Using optogenetic perturbation to selectively silence auditory cortex during early noise processing, we show that cortical processing plays a crucial role in the segregation of competing sounds.

## Introduction

In the acoustic world, animals are challenged to detect salient sounds in noisy backgrounds, a process of critical importance in communication, hunting, and threat-detection. The auditory system is well suited to the task as it demonstrates a remarkable spectral (Rosenblith and Stevens, 1953) and temporal resolution (Klumpp and Eady, 1956; Plomp, 1964), and this acuity is valuable for detecting changes in natural soundscapes (McDermott et al., 2013; Andreou et al., 2015). Predictable, or coherent, nonrandom features of soundscapes may be exploited by the auditory system to improve sound processing (Ulanovsky et al., 2003; Taaseh et al., 2011; Yaron et al., 2012; Teki et al., 2013; Krishnan et al., 2014; Nelken, 2014). A prevalent feature of natural sound is coherent amplitude fluctuations across frequency, termed “comodulation” (CM). CM is present in both environmental sounds and vocalizations (Nelken et al., 1999). Given its pervasiveness in nature, CM may be a critical cue for grouping and segregating overlapping sounds (Nelken et al., 1999; Krishnan et al., 2014).

Comodulation masking release (CMR) is a psychoacoustic phenomenon whereby adding coherently modulated noise to an existing masker makes signals easier to perceive (Hall et al., 1984). This effect is striking as additional noise energy normally reduces, or does not change, signal detectability (Fletcher, 1940). CMR encompasses two separate processes, dependent on the relative frequencies of the noise and the signal: within-channel CMR (signal and noise similar in frequency) and across-channel CMR (signal and noise dissimilar in frequency). Within-channel CMR can be implemented in the auditory periphery, but across-channel or “true” CMR (Verhey et al., 2003) cannot be explained by mechanical processes in the ear and is sensitive to cues of auditory grouping (Buss et al., 2009; Dau et al., 2009; Verhey et al., 2012). Across-channel CMR is therefore a result of brain processing, but the mechanism and location of such processing are not well understood.

Only a small number of studies have directly sought to understand the representation and underlying mechanism(s) of CMR at the cellular level. In the peripheral auditory system, neuronal responses to pure tones are enhanced by across-channel CM in a way consistent with human behavior (Pressnitzer et al., 2001). However, it is not clear whether this information is inherited or influenced by processing at later stages of the auditory system. A CMR correlate has been shown to develop progressively between the inferior colliculus, medial geniculate body, and auditory cortex (Nelken et al., 1999; Las et al., 2005), although this work explored both within- and across-channel cues simultaneously. As such, it remains unclear how much of the observed CMR is attributable to across-channel processes (Verhey et al., 2003; Grose et al., 2005a). Neuronal correlates of within-channel CMR have been observed in the avian auditory forebrain area L2a; however, when measured in an across-channel configuration (comparing narrowband [NB] and broadband comodulated noise), no significant CMR was found (Nieder and Klump, 2001; Hofer and Klump, 2003).

In this study, we set out to quantify the influence of across-channel CM on signal detectability in the neuronal activity of primary auditory cortex (A1), a critical site in auditory perception (Bizley and Cohen, 2013). We then sought to establish whether processing by auditory cortical circuits plays a functional role in the formation of across-channel CMR. We provide the first quantification of across-channel CMR in A1. Transient inactivation during early sound processing significantly reduces subsequent masking release, suggesting that auditory cortex may play a causal role in enhancing signal representation in the presence of comodulated noise.

## Materials and Methods

### 

#### 

##### Animals and preparation.

The care and experimental manipulation of animals were performed in accordance with institutional and United Kingdom Home Office guidelines. Homozygous *Pvalb*-IRES-Cre mice (JAX stock #008069) of both genders were used in this study. At 6 weeks, auditory cortex was injected unilaterally with AAV-EF1a-DIO-hChR2(H134R)-EYFP virus to express selectively channelrhodopsin (ChR2) and EYFP proteins in parvalbumin-positive (PV^+^) interneurons. Mice were anesthetized with 1%–2% isoflurane under aseptic conditions and held using ear bars on a stereotaxic mount (Angle 2, Leica). A small burr hole was made with a dental drill ∼1 m lateral from midline and ∼2.7 mm caudal to bregma. A small glass pipette holding the virus was advanced to reside in auditory cortex; 0.5 μl was then slowly injected into cortex over a period of 15 min. The pipette was then removed and the burr hole sealed with Kwik-Cast (World Precision Instruments), once dry acrylic dental cement was layered over the top forming a hard seal over the site. The area was then cleaned (iodine) and the tissue sealed (Histoacryl, Braun). Analgesia was administered during the procedure via intraperitoneal injection (Carprofen; 5 mg/kg). The animal was then recovered and the virus left to express for 2 weeks, during which time buprenorphine (0.8 mg/kg) jelly was used for postoperative analgesia.

##### Electrophysiology.

For electrophysiological recordings, mice were anesthetized with a fentanyl/midazolam/medetomidine/mixture (0.05, 5.0, and 0.5 mg/kg). A midline incision was made, and all tissue was cleared from the cranium. Hatched score lines were made on the cranial surface with a dental drill to increase surface area and a customized headplate was fixed using adhesive (Histoacryl, Braun). A small craniotomy was made directly above auditory cortex, and dura was removed using fine forceps. Mice were fixed by clamping the headplate to a customized clamp stand. A silicon microelectrode (A32, 4×2Tet Neuronexus) was advanced via micromanipulator (IVM, Scientifica) into auditory cortex at an angle perpendicular to the cortical surface. An optical fiber, attached to DPSS laser (473 nm, 100 mW; SLOC), was positioned between the middle two probe shanks ∼1 mm from the cortical surface. The laser was controlled via TTL signals generated via digital processor (RZ6, Tucker Davis Technology). A number of laser durations were trialed, with the intention of disrupting cortical activity for the period of the precursor (0–400 ms) but not disrupting cortical activity to the later masker (500–1000 ms). We found that laser duration of 150 ms (from 0 to 150 ms) was sufficient to produce transient suppression (see Fig. 6*C*).

Data were acquired via Digital Lynx 16SX system (Neuralynx) and stored on a PC. Recordings were made in primary auditory cortex from a total of 21 sites at different penetrations, in 6 animals.

##### Auditory stimulation.

Auditory stimuli were generated and calibrated using MATLAB (The MathWorks) and stored as files to be used when required. All signal levels were calibrated (5–100 kHz flat spectrum ±1.5 dB SPL). Stimuli were sinusoid amplitude modulated (SAM) tone maskers presented in the presence of a pure tone signal. Three bandwidth configurations were used: a NB condition (10 Hz SAM 20 kHz pure tone) and two broadband conditions (incoherent modulation [IM] and CM). The broadband conditions consisted of an on-frequency band (10 Hz SAM 20 kHz pure tone), low off-frequency bands (3 × 0.125 octave spaced 10 Hz SAM pure tones centered at 12.9 kHz), and high off-frequency bands (3 × 0.125 octave spaced 10 Hz SAM pure tones centered at 30.8 kHz). Off-frequency bands were designed so that all components fell outside a mouse auditory filter (May et al., 2006). For all conditions, the phase of the envelope of the on-frequency band remained identical. In the IM condition, the envelope of the off-frequency bands was selected at random between 0° and 180°; and in the CM condition, the off-frequency bands had identical envelope phases with the on frequency band. The NB, IM, and (long-) CM conditions were all composed of a precursor (400 ms) and masker portion (500 ms) with a short gap in between (100 ms). A short-CM condition was also used; this was identical to the long-CM condition but without the precursor. An interstimulus interval of 2 s was used. The signal was comprised of three 50 ms SAM tone pips (10 Hz modulator), occurring in the 3rd, 4th, and 5th troughs of the masker. Eight different signal conditions were used for each masker condition, a noise alone (i.e., no signal) condition and seven noise + signal conditions (−10 to 20 dB signal-to-noise ratio [SNR] in 5 dB steps, where the masker level was held at 65 dB SPL and the signal level varied).

Frequency response areas (FRAs) were measured using pure tones, at 25 different frequencies (500 ms duration, 7–57 kHz, with 0.25 octave spacing), 8 different sound levels (10–80 dB SPL at 10 dB steps), with a 1 s interstimulus interval. Stimuli for both FRA and masking experiments were presented in blocks, a single repeat of each stimulus combination was presented in each block (frequency/level or masker signal combination, respectively). The order of the stimuli within a block was randomly selected. Blocks of masking condition and FRA stimuli were interleaved such that five blocks of masking condition were followed by one block of the FRA stimuli. In total, there were 30 repeats of each masker/signal combination and 6 repeats of each frequency/SPL combination. All stimuli were saved in the above structure into a single file to be used for neuronal recordings. Sound files were presented using MATLAB to interface with RPvdsEX (Tucker Davis Technology) running on an RZ6 (Tucker Davis Technology) driving a free-field speaker (ES1, Tucker Davis Technology).

##### Data analysis.

Spikes were extracted from the raw data files using SpikeDetekt (http://sourceforge.net/projects/spikedetekt/) and initially clustered using KlustaKwik. Clusters were manually inspected using Klusters and reclustered when necessary, clusters that contained >1% of spikes within a 1 ms interspike interval were rejected (http://neurosuite.sourceforge.net). Spike time data were extracted and exported to MATLAB for further processing.

Raw FRAs were initially smoothed using a 3 × 3 pyramidal window; iso-response curves (FRA edges) were then determined by finding a 30% change from baseline firing rate (Sutter and Schreiner, 1991; Scholes et al., 2011). Both excitatory (positive criterion) and inhibitory (negative criterion) curves were defined but processed separately. Each curve circumscribed a defined region. Inspection of the resulting defined regions revealed that using a single criterion meant that two strong responsive regions, separated by a weak response in between, could be defined as two independent regions (because the weak region did not cross criterion). To prevent this, an additional stage was added where a 15% criterion was used and regions connected by this lower criterion were grouped into single regions (regions were defined using *bwboundaries* command in MATLAB, excitatory regions were only grouped with other excitatory regions). The largest defined region was always used for further analysis. Characteristic frequency was taken as the frequency yielding a defined response at the lowest signal level.

Significant changes in firing rate of two conditions were detected by pooling the single presentation repeats of those conditions and bootstrapping to produce a distribution of mean firing rate (500 samples, sample with replacement). This distribution was then used to define significant increases in firing rate (mean firing rate *p* > 0.95). To compare responsiveness to the signal and ignore the plethora of responses to the noise, we calculated the following signal response:

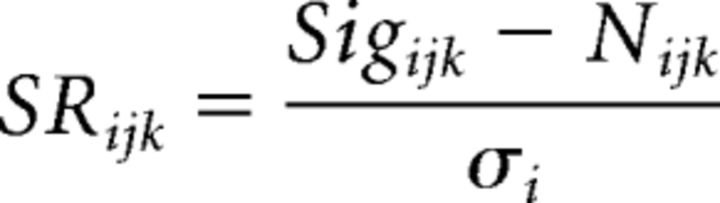
 Where *SR* is the signal response peristimulus time histogram (PSTH), *Sig* the noise and signal PSTH, and *N* the noise alone PSTH (5 ms bins were used for all PSTHs) and where *i* is the cell number, *j* the background noise condition index, and *k* the SNR index. σ was calculated as the SD of all possible firing rates for that cell (i.e., all bins from all PSTHs for that cell were included in this measure). The SR PSTH for each condition was then averaged between 1.175 and 1.275 s (i.e., the period of the first tone pip). This produced a SR/SNR function for each cell, which was upsampled (linear interpolation) in the SNR dimension and the individual cell threshold estimated (0.5 SD criterion crossing).

The population signal response was the mean of the individual signal responses. As before, this was then averaged between 1.175 and 1.275 s to produce the mean SR/SNR function. To compare signal sensitivity and tuning properties we grouped cells, based on their characteristic frequency (CF), and data were bootstrapped to allow comparison across populations (50 selections per subpopulation with replacement, 500 repeats). This gave 500 SR/SNR functions, the mean of which was used to estimate the SR/SNR function for each subpopulation. An arbitrary criterion of 0.25 SD was selected; this value was intentionally low to allow subpopulations with only weak responses to be included in this analysis. To quantify thresholds, firing rates were converted into a neurometric function for each individual condition, by comparing the mean firing rate to the noise alone versus each SNR measured. Each cell was given a “vote” to respond whether that cell detected a signal. A relative increase in firing rate (≥25%) was equivalent to a “yes” (and given a value of 1), a decrease (≥25%) was equivalent to a “no” (a value of 0), and anything in-between was set to chance (a value of 0.5). The population proportion correct was the mean value across the population. This neurometric function (proportion correct vs SNR) was upsampled (linear interpolation) and binomial probability used to estimate threshold (above chance performance, *p* < 0.05 Bonferonni corrected).

To quantify the effect of band-widening on the onset responses, we calculated across-frequency interaction (AFI; i.e., the change in onset response between the NB and broadband conditions). Across the population, cortical firing rates were distributed in a log-normal fashion (Buzsáki and Mizuseki, 2014). Thus, to allow comparison of changes in spike count across our population, we converted spike counts to a linear scale (exponential function) and then normalized by dividing by the maximum response of a given cell across all of the stimuli presented. Using these normalized spike counts, AFI was calculated by subtracting the total spikes, within the onset window (0–75 ms), in response to the NB stimuli from the response to the broadband stimuli (IM and CM, respectively). Therefore, positive values reflect facilitation in spiking, relative to the NB condition, and negative values reflect suppression.

All data are presented as mean ± SEM unless otherwise stated.

## Results

### Signal- and noise-evoked activity in primary auditory cortical populations

**Figure 1.**
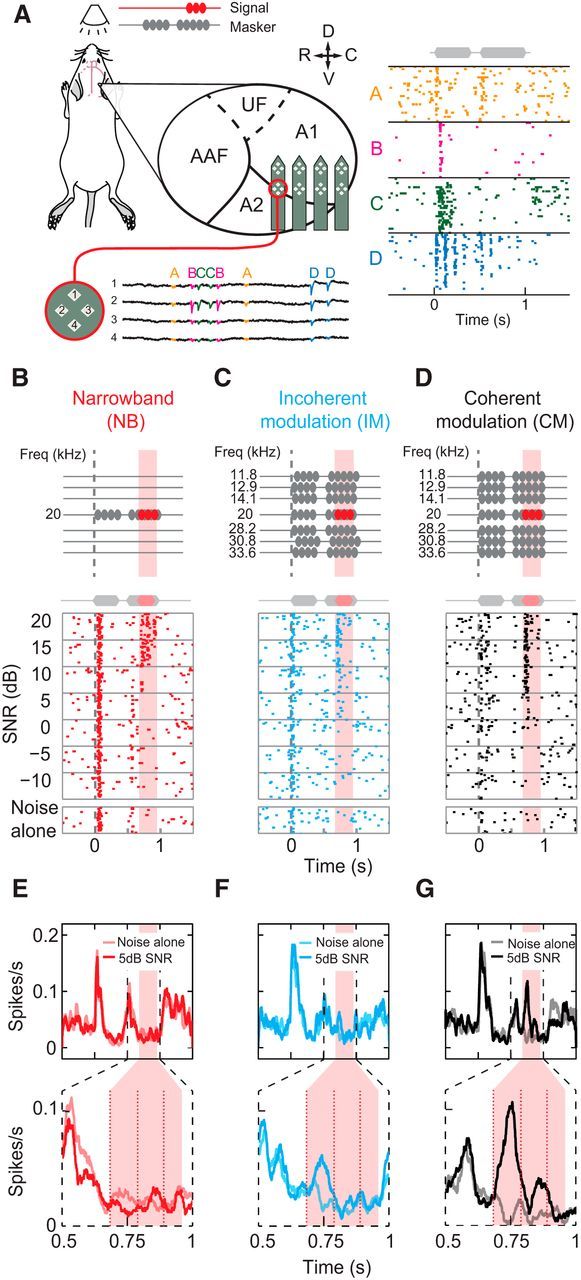
Signal-evoked activity in mouse primary auditory cortex in the presence of masking noise. ***A***, Experimental setup. Recordings were made from primary auditory cortex (A1) using 4 × 2 tetrodes. Extracellularly recorded action potentials (spikes; bottom left) were sorted to isolate evoked single neuron activity (right). ***B–D***, Pure tone signals were presented in three different noise backgrounds: (***B***) NB (red), (***C***) broadband with IM (blue), and (***D***) broadband with comodulated envelopes (CM, black). ***B–D***, Bottom, Raster plots of evoked spiking activity of a single cell. Each mark indicates a spike (30 consecutive trials for each condition). Noise was presented alone and in the presence of three tone pips at different SNRs (ordinate). Gray dashed lines indicate noise onset. Pink backgrounds represent the tone presentation window. ***E–G***, PSTHs of the same cell for noise alone (light colored lines) and 5 dB SNR (dark colored lines) stimuli for each masking condition (NB, IM, and CM, respectively). Dashed line indicates the time period around the signal (top row) and is magnified (bottom row). Red dashed lines indicate onset of individual tone pips.

Our first aim was to establish whether neuronal correlates of across-channel CMR are present in individual neurons of A1 (Linden et al., 2003). Simultaneous extracellular population recordings were made in anesthetized mice (Fig. 1*A*), and evoked activity was measured during the presentation of a pure tone signal (train of 20 kHz tone pips) embedded in masking noise at a variety of SNRs (range: − 10 to 20 dB, 5 dB steps, masker level fixed at 65 dB SPL). Across the population, evoked responses were observed to both signal and masker, including a wide variety of firing profiles to the masking noise alone (Fig. 1*B–G*). Psychoacoustic CMR is characterized by improved signal detectability produced by adding energy to an existing masker (e.g., a NB noise). It is observed when (1) extra noise energy is added away from the signal frequency (i.e., increasing the bandwidth); and (2) the envelope of this noise energy is coherently modulated with the NB masker. We expected that both conditions should be met to observe large masking release of signal-evoked neuronal activity. Additional noise energy, in the form of amplitude-modulated “flanking bands” of pure tones, was therefore added to the masking stimulus (6 pure tones split into low- and high-frequency flanking bands; see Materials and Methods). The modulator phase of flanking noise bands was presented either incoherently (IM; Fig. 1*C*), or coherently (CM; Fig. 1*D*), with respect to the NB masker (Fig. 1*B*). Thus, by comparing signal-evoked responses between three distinct noise conditions (NB, IM, CM; Fig. 1*B–G*), we measured the influence of across-frequency coherence on signal detectability in A1.

Most cells demonstrated a simple relationship between spiking and SNR, whereby increased signal levels evoked more action potentials (Fig. 1*B–D*). The majority of cells only responded to the first of the three tone pips, although examples were found of cells responding to the entire train. In individual cortical neurons, we found only small differences in signal-evoked responses between NB and broadband IM maskers (Fig. 1*B*,*C*,*E*,*F*). Therefore, adding sound energy to a masking noise by itself neither impaired nor improved the detectability of signals at low SNRs when this energy was restricted to frequencies away from the signal. However, coherent modulation of flanking noise bands produced large enhancements in signal-evoked activity, consistent with psychoacoustic measurements and corresponding to a true release from masking (Fig. 1*D*,*G*).

### Selective enhancement of neuronal sensitivity via CM

**Figure 2.**
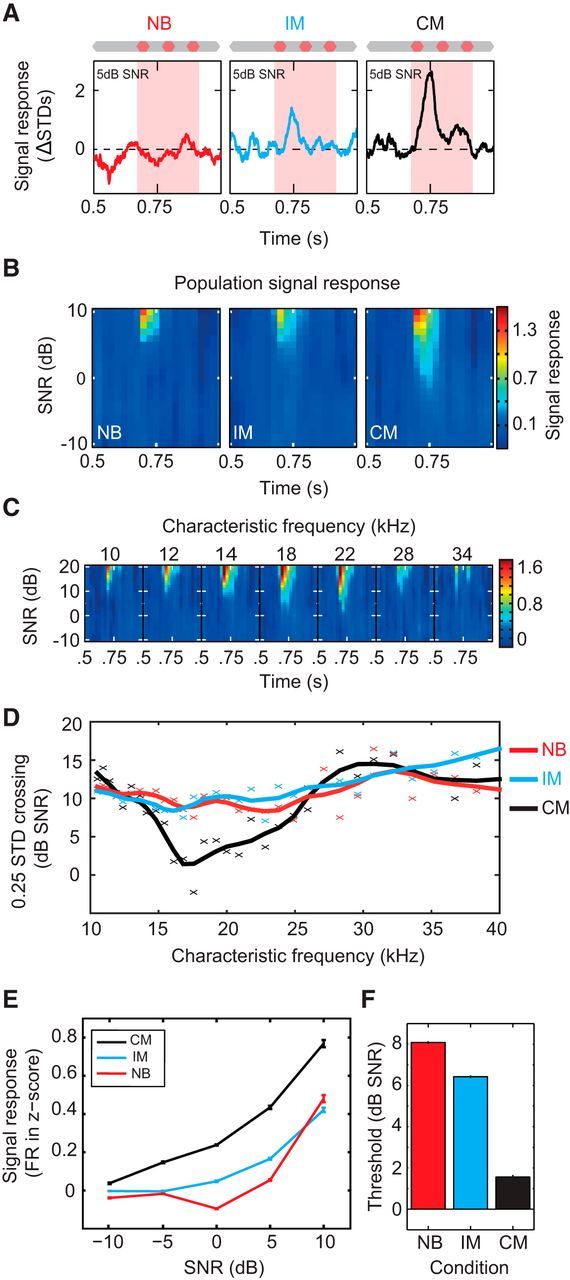
Comodulated flanking noise produces large masking release in signal-tuned neurons. ***A***, Signal-evoked response of individual cell (shown in Fig. 1*B–G*) in NB, IM, and CM backgrounds at 5 dB SNR (left to right). ***B***, Population mean signal response. The mean signal response for the NB, IM, and CM (left to right) conditions was calculated across all cells demonstrating a significant signal response (n_NB_ = 887, n_IM_ = 842, and n_CM_ = 797). ***C***, The effect of frequency selectivity on signal sensitivity. Mean signal response in CM noise for subpopulations grouped by CF (see Materials and Methods). ***D***, Signal sensitivity of CF-defined subpopulations in different masking conditions. The sensitivity of each subpopulation was defined using 0.25 SD change in firing rate as a criterion. Lines represent linear interpolation of rolling average (3 data points). ***E***, Population (n_tuned_ = 499) signal response function in NB, IM, and CM backgrounds. ***F***, Population detection thresholds. Cell responses were converted into a population neurometric function from thresholds that were estimated using binomial probability (*p* < 0.05 Bonferonni corrected).

To isolate signal-evoked activity, the mean spiking response to a masked signal was subtracted from the response to the isolated masker (noise alone; Fig. 2*A*). Salient features of auditory scenes are encoded by the concerted activity of neuronal ensembles (Harris et al., 2011; Bathellier et al., 2012). Signal sensitivity across A1 was assayed by calculating the population mean signal response, this included all cells that demonstrated significant signal responses to at least one SNR/noise combination (n_all_ = 1271; Fig. 2*B*). Cortical signal detectability was compared across different masking backgrounds. The minimum sound level at which signal-evoked responses were observed (i.e., the overall cortical sensitivity) was similar in NB and IM conditions (Fig. 2*B*; NB vs IM), whereas responses in coherently modulated broadband noise were detected at markedly lower SNRs (Fig. 2*B*; NB or IM vs CM). Frequency channels in A1 therefore remain relatively independent of one another during the presentation of coincident, but incoherently modulated, sounds. Coherent noise modulation is necessary for masking release at the signal frequency, suggesting that interactions across sound frequencies are necessary to improve cortical sensitivity to signals at low SNRs.

Having established that neuronal correlates of across-channel CMR are present in A1, we sought to identify whether particular subsets of neurons are responsible for the overall improvement in signal representation. Frequency selectivity is a fundamental property of auditory cortical neurons and is a key parameter in determining individual neuronal sensitivity to tonal signals. We expected that the most robust signal-evoked responses would be from neurons tuned at or close to the 20 kHz signal frequency. The overall increase in signal-evoked responses in comodulated noise could result from enhanced sensitivity of this subset of neurons whose selectivity is centered on the signal frequency. Alternatively, increased sensitivity at the population level could be a consequence of additional recruitment of neurons whose receptive fields are centered away from the signal frequency. When sorted according to CF (each cell's most sensitive frequency between 7 and 56.6 kHz; see Materials and Methods), cells tuned to the signal frequency demonstrated the highest sensitivity (Fig. 2*C*) and the largest masking release in CM versus IM/NB maskers (Fig. 2*D*). Thus, increases in sensitivity to the signal were largely restricted to neurons tuned to frequency bands adjacent to, and encompassing, the signal frequency. Large differences in sensitivity were observed between the most and least sensitive subpopulations in all three masking conditions (CM: 15.7 dB SNR; NB: 8.28 dB SNR; IM: 6.12 dB SNR). These results demonstrate that improvements in signal detectability at the population level are accounted for by neurons with receptive fields centered at, or close to, signal frequency, and that improvements in detectability afforded by CM are restricted to this same population (i.e., signal-selective neurons).

We compared the response functions of signal-selective neurons (CF = 15–23 kHz, n_tuned_ = 499) in different masking environments. As expected, the properties of the masker had a significant effect on signal-response functions (Friedman test, χ_(2)_^2^ = 172.9, *p* < 0.01). Incoherent flanking noise produced relatively small increases in firing rate at low SNRs relative to the NB condition, although this difference was significant (Fig. 2*E*; NB vs IM, Wilcoxon rank sign test, *Z* = −5.4, *p* < 0.01). By contrast, comodulated flanking noise produced significantly larger changes in firing rate than both NB and IM conditions (Wilcoxon rank sign test, CM vs NB and CM vs IM, *Z* = 15.4 and 12.5, respectively, *p* < 0.01). In psychophysics, CMR is a reduction in the perceptual thresholds of participants. We tested whether the neuronal limits of signal representation resembled the limits of perception. To this end, a population-based neurometric function was calculated from the single-cell data for each condition (Fig. 2*F*) and from this function thresholds were derived. Threshold was defined as the SNR at which neurometric performance was above chance (binomial probability; see Materials and Methods). Incoherent flanking noise was associated with a small (1.7 dB) but significant reduction in threshold (NB vs IM, 8.1 vs 6.4 dB SNR, 2 independent-sample *t* test, *p* < 0.01), whereas CM of flanking noise produced a much larger (6.6 dB) significant reduction in threshold (NB vs CM, 8.1 vs 1 dB SNR, 2 independent-sample *t* test, *p* < 0.01). Thus, although adding flanking noise did produce a significant change in threshold, this change was relatively small (∼4 times smaller), and CM of this noise is necessary for large reductions in signal detection threshold within A1.

### Across-frequency coherence alters early noise processing and predicts strong masking release

**Figure 3.**
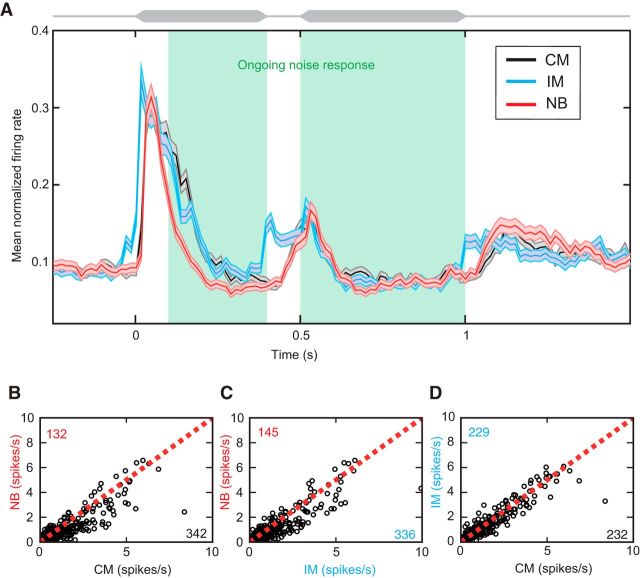
Broadband noise facilitates ongoing noise-evoked responses. ***A***, Normalized firing rates of cells tuned close to the signal frequency (15–23 kHz; n_tuned_ = 499) for noise-only conditions. Black represents CM. Blue represents IM. Red represents NB. ***B***, Comparison of individual firing rates (averaged from between 0.1 and 0.4 and 0.6–1 s) for the CM and NB conditions. Red dashed line indicates when the firing rates are matched. Value in the top left corner = number of points falling above the line; bottom right = number of points falling below the line. ***C***, Comparison of individual firing rates for the IM and NB conditions. ***D***, Comparison of individual firing rates for the IM and CM conditions. Adding spectral energy facilitates ongoing noise responses regardless of temporal features of the masker.

Our results reveal that interactions across sound frequency can alter sensory representations in A1, and that changing the temporal characteristics of the noise alone can enhance signal-evoked activity. Little is known about the neuronal mechanisms that underlie across-channel CMR, but psychophysical models have suggested that ongoing cancellation of the noise at the signal frequency might be the cause of improvements in detectability (Piechowiak et al., 2007). Such a process could be implemented by lateral inhibitory mechanisms upstream of, or within, A1. Under this scheme, neurons tuned to the signal frequency are predicted to exhibit a relative decrease in ongoing noise-evoked activity for CM versus NB maskers. We compared ongoing (100–400 ms and 500–1000 ms) noise-evoked responses in the three background conditions for the signal selective cells (CF = 15–23 kHz, n_tuned_ = 499). Surprisingly, increasing bandwidth produced a significant increase in firing rate for both broadband (CM and IM) conditions relative to the NB condition across this population (Fig. 3; sign test, CM, *p* ≪ 0.01, IM, *p* ≪ 0.01). Furthermore, in individual neurons, differences between CM and NB noise-evoked responses during the signal window were not correlated with masking release (Pearson's Product-moment correlation; PPMC, *r* = 0.048, *p* = 0.36). This demonstrates that there is no evidence of active cancellation (or reduction) of neuronal responses to noise at the level of cortex and the magnitude and relative change of noise-evoked activity in CM versus NB conditions during the signal window were not predictive of signal sensitivity. The effect of coherent and incoherent modulation on the ongoing representation of noise responses was also contrasted (Fig. 3*D*). There was no significant difference in spiking responses to the noise between coherent and incoherent modulation, suggesting that increases in firing rate were spectral and not related to modulation or across-channel processes (Fig. 3*D*; sign test, *p* = 0.93).

**Figure 4.**
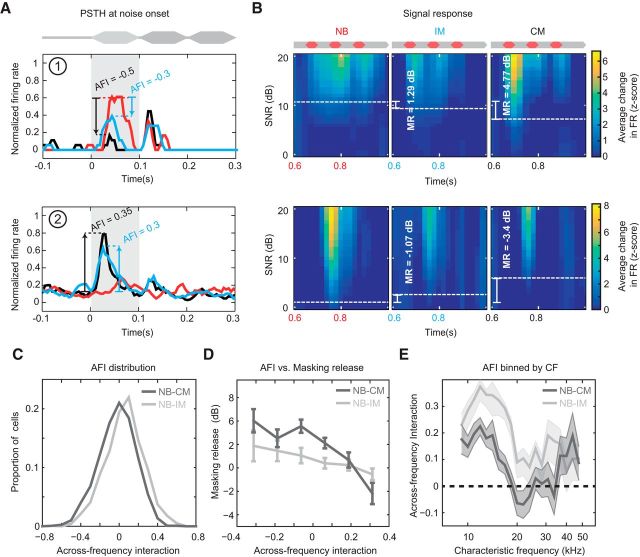
Early noise responses are predictive of masking release. ***A***, Noise-evoked responses from two example cells demonstrating different AFIs when comparing between NB and broadband noise conditions. Red line indicates the response to the NB noise. Blue line indicates the response to broadband IM noise. Black line indicates the response to broadband CM noise. Hatched lines indicate differences in evoked response between NB and broadband conditions (NB → IM and NB → CM; blue and black AFI values, respectively; see Materials and Methods). ***B***, Signal response functions for the same cells in different masker conditions. Mean firing rate changes were used to derive thresholds for each condition (dashed white lines). Differences in thresholds correspond to masking release (MR) produced by adding broadband comodulated bands, indicated in each panel. ***C***, AFI distribution for all cells in IM (light gray) and CM (dark gray) conditions. CM is associated with negative AFI values (i.e., reduced evoked activity to noise onset). ***D***, Relationship between AFI and masking release. Individual cells were binned based on their AFI. Negative AFIs values are associated with increased masking release in broadband maskers. ***E***, Relationship between AFI and frequency tuning. Neurons tuned at or close to signal frequency exhibited negative AFIs in the presence of broadband CM but not IM noise.

Psychophysical experiments indicate that properties of the early portion of the background noise, such as onset synchrony, duration, and spectrotemporal statistics, are critical in the formation of a large across-channel CMR (McFadden and Wright, 1990, 1992; Grose and Hall, 1993; Dau et al., 2005; Grose et al., 2009; Verhey et al., 2012). We therefore looked for evidence of a role for early noise processing in masking release at the level of A1. Initially, we compared the relative amplitude of the early periods (first 100 ms) of broadband (IM and CM) and NB noise-evoked responses for individual neurons, and found marked differences in neuronal firing rates between onset responses for each noise conditions. Evoked responses to broadband noise were often either suppressed or facilitated relative to NB noise (Fig. 4*A*). Differences between CM and NB noise-evoked responses were quantified with a metric we termed Across Frequency Interaction (AFI), where positive values indicated facilitation and negative values indicated suppression in noise-evoked activity to the onset in the broad relative to narrow-band conditions (Fig. 4*A*,*B*; see Material and Methods). We investigated the relationship between AFI and masking release in all cells demonstrating significant responses to the signal (n_all_ = 1271). The addition of the flanking noise tended to facilitate NB noise onset responses, demonstrated by slightly positive, yet significant, mean AFI values (mean CM AFI = 0.0284, sign test, *p* ≪ 0.01, IM AFI = 0.1029, sign test, *p* ≪ 0.01). However, the type of background masker had a significant effect on AFI where CM significantly shifted the distribution of AFI values in the negative direction (Fig. 4*C*; K-S test, D = 0.1599, *p* ≪ 0.01). Interestingly, when we compared AFI values with CMR measurements in individual neurons (see Materials and Methods), we observed a clear pattern: cells demonstrating suppressed onset responses to broadband noise (i.e., negative AFI) demonstrated strong masking release (Fig. 4*A*,*B*, top row). Cells with enhanced broadband onset responses (i.e., positive AFI) showed the opposite behavior (an overall increase in masking, Fig. 4*A*,*B*, bottom row). Plotting AFI and masking release demonstrated suppression during noise onset was associated with masking release, AFI varied significantly with masking release for the CM condition (ANOVA, *F*_(5)_ = 3.73, *p* = 0.0024) but not for the IM condition (ANOVA, *F*_(5)_ = 2.15, *p* = 0.0574). In addition, a significant correlation between AFI and masking release was found in single units for the CM condition (Fig. 4*D*; PPMC, *r* = −0.12, *p* ≪ 0.01) but fell short of significance for the IM condition (PPMC, *r* = −0.059, *p* = 0.067). Therefore, at least for the CM condition, early noise responses (quantified here with AFI) were predictive of subsequent masking release. Finally, the relationship between AFI and CF was investigated. AFI varied significantly with CF for both broadband masking conditions (MANOVA, *p* ≪ 0.01). *Post hoc* tests revealed both CM (ANOVA, *F*_(23)_ = 2.22, *p* = 0.0008) and IM (ANOVA, *F*_(23)_ = 4.18, *p* ≪ 0.01) varied significantly with CF. For the CM background, the AFI was lowest close to the signal frequency (∼20 kHz), where it became negative (i.e., suppressive). Comparison of noise onset firing rates in the NB and CM condition confirmed that this suppression was significant (sign test, 20 kHz, *p* = 0.0172 and 21.8 kHz, *p* ≪ 0.01). Those cells exhibiting the greatest masking release (those tuned at, or close to the signal frequency; Fig. 2*C*) also demonstrated the strongest onset suppression to CM versus NB noise (Fig. 4*E*). Altered responses during early sound processing are therefore a prominent feature of neurons that exhibit strong masking release, and the across-frequency suppression of noise-evoked onset responses is positively related to CMR.

### Priming significantly enhances CMR in auditory cortex

Across-channel CMR is affected by the temporal history of the masker (McFadden and Wright, 1992; Grose and Hall, 1993; Dau et al., 2005; Grose et al., 2005b, 2009; Verhey et al., 2012). Increasing the preceding duration of comodulated noise enhances signal detection (McFadden and Wright, 1990, 1992; Hatch et al., 1995); and when comodulated noise preceding and following the signal segment is replaced with random noise, the CMR effect is cancelled (Grose et al., 2005b, 2009). Furthermore, onset asynchrony of noise across frequency prohibits CMR (McFadden and Wright, 1992; Grose and Hall, 1993; Verhey et al., 2012). Together, these findings demonstrate the importance of the history of a noise masker and imply that the detectability of quiet signals is greatest when the auditory system has been primed or adapted to these features of the noise. The prevalence of such higher-order influences on across-channel CMR suggests an important role for higher-order processing, such as may occur in auditory cortex.

**Figure 5.**
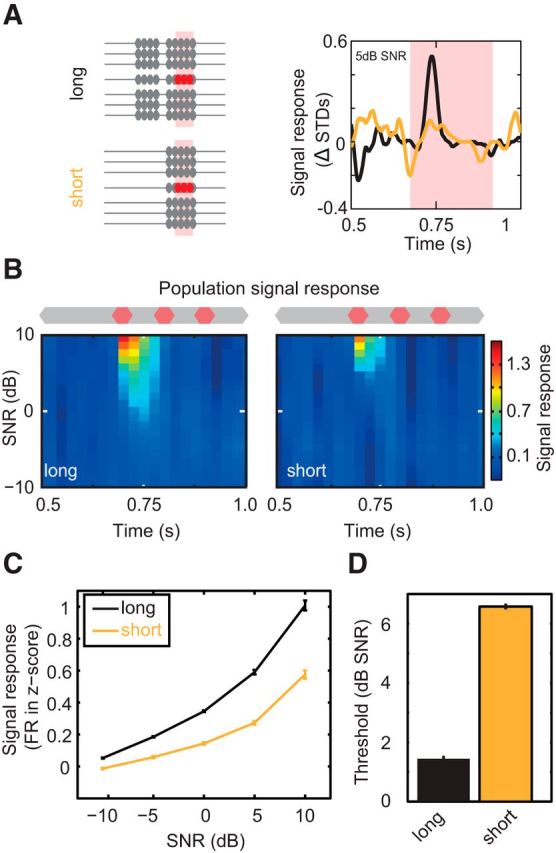
Priming noise exposure is necessary for large masking release. ***A***, Signal-evoked responses in short (yellow) and long (black) maskers. ***B***, Population mean signal response for short- and long-CM-noise conditions (n_tuned-CM_ = 358). ***C***, Population signal response function. ***D***, Population detection thresholds. Cell responses were converted into a population neurometric function from the thresholds estimated using binomial probability (*p* < 0.05 Bonferonni corrected).

Having observed a close relationship between early processing of masking sounds and across-channel CMR, we sought to functionally test the importance of the initial noise-processing period on signal detection in A1. We hypothesized that exposure to the early noise portion (between 0 and 400 ms) primes the auditory system to the spectrotemporal statistics of the masker to facilitate masking release. We therefore measured the impact of prolonged exposure to the spectrotemporal statistics of the masker (i.e., priming) on the magnitude of across-channel CMR. We examined this by removing the early portion of the noise and measuring the effect on signal detectability in tuned cells sensitive to CM (n_tuned-CM_ = 358). Priming had a large impact on signal sensitivity, demonstrated by large decreases in the population signal when changing from a long to a short-CM masker (Fig. 5*A*,*B*). This equated to a significant decrease in the signal response function (Fig. 5*C*; Friedman test, χ_(2)_^2^ = 68.15, *p* ≪ 0.01), and decrease in masking release. The neurometric threshold decreased significantly by 5.6 dB (Fig. 5*D*; from −0.2 dB SNR to 5.3 dB SNR, 2 independent-sample *t* test, *p* ≪ 0.01), demonstrating that preceding exposure to comodulated noise enhances subsequent across-channel masking release. Priming is therefore necessary to induce large across-channel CMR in A1.

**Figure 6.**
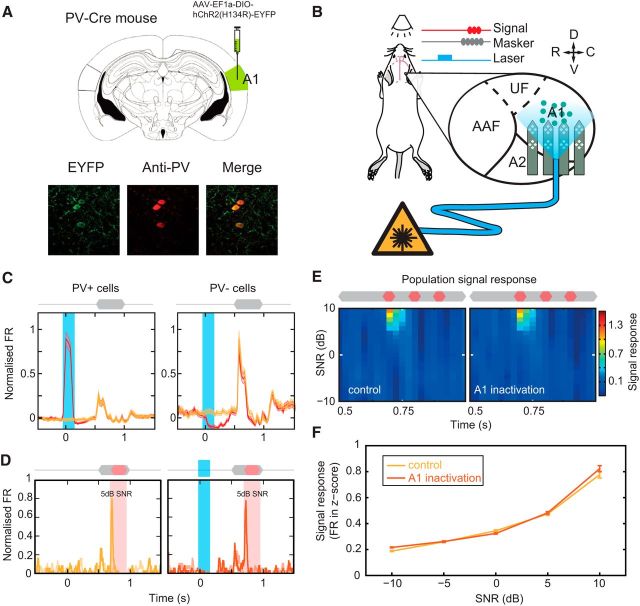
Preceding cortical inactivation does not influence subsequent signal-evoked responses. ***A***, Top, AAV-EF1a-DIO-hChR2(H134R)-EYFP was injected into A1 of *Pvalb*-IRES-Cre mice. Bottom, Specific infection of PV^+^ cells was confirmed via coexpression (merged image, right) of ChR2 (green, left) and PV^−^ antibody (red, middle). ***B***, Optogenetic inactivation of auditory cortex before presentation of noise and signal. Schematic showing approach to functionally measure the role of cortical early sound processing via transient selective inactivation of A1. Activation of ChR2-expressing PV^+^ interneurons (teal circles) was used to inhibit ongoing cortical activity. ***C***, Brief (150 ms) laser stimulation of A1 induced strong activation of PV^+^ cells (left) and inhibition of PV^−^ cells (right). The fidelity of the approach was confirmed by comparing population PSTH responses with noise presentation in the presence (red) or absence (yellow) of preceding laser stimulation. While cortical activity was strongly modulated during laser stimulation, subsequent auditory-evoked responses were unchanged in both PV^+^ and PV^−^ cells. ***D***, Signal-evoked responses from PV^−^ cells were tested using brief CM noise maskers in the absence (control, yellow) and presence (A1 inactivation, red) of preceding laser stimulation. ***E***, Laser stimulation did not influence the population signal response (n_tuned-CM_ = 358). ***F***, Population signal response function under the two conditions. No significant differences were observed.

**Figure 7.**
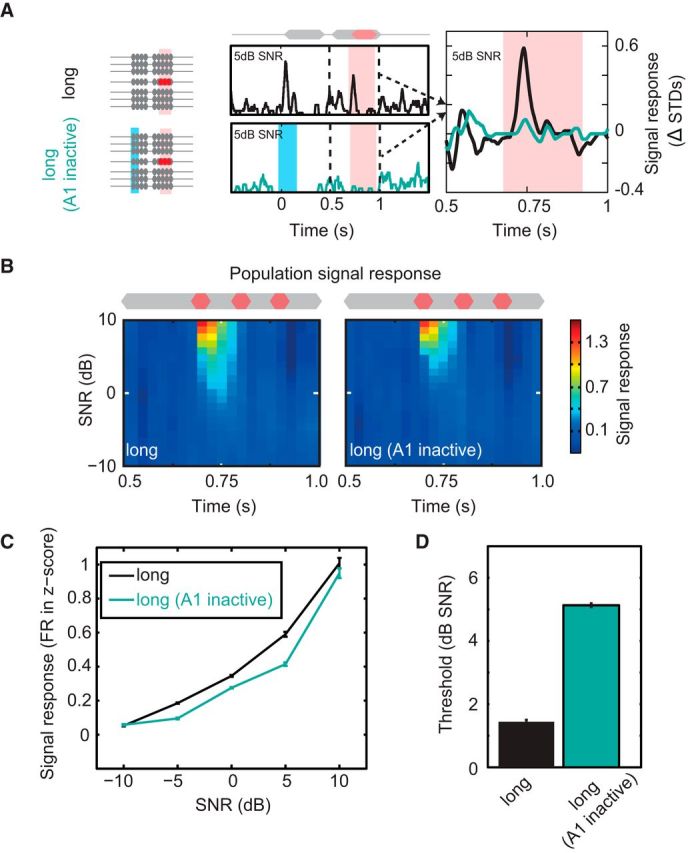
Processing of the early noise is required for large masking release in auditory cortex. ***A***, The role of A1 in across-channel CMR was tested by inactivating A1 during the early noise period (left, long; A1 inactive). Blue bar represents laser on. Middle, Single-cell example PSTHs for the normal (top, long, black line) and silenced (bottom, long; A1 inactive, teal) conditions. Right, Signal response for during long-CM masker, with (teal) and without (black) preceding A1 inactivation. ***B***, Population mean signal response for long, and long-A1 inactive conditions (n_tuned-CM_ = 358). ***C***, Population signal response function. ***D***, Population detection thresholds. Cell responses were converted into a population neurometric function from the thresholds estimated using binomial probability (*p* < 0.05 Bonferonni corrected). Cortical inactivation during early presentation of the noise reduced improvements in signal detection thresholds.

At what stage of the ascending auditory system does priming to spectrotemporal statistics occur? This process could occur exclusively via subcortical mechanisms in which case the improvements in signal detectability will be passively inherited in the inputs to A1. Alternatively, A1 could itself play a role in priming to ongoing noise to enhance signal detection (e.g., via local circuit mechanisms and/or feedback to subcortical stations). We therefore set out to assess whether cortex plays a direct role in the formulation of large across-channel CMR. To do this, we applied a paradigm to transiently and specifically disrupt neuronal activity in A1 during early sound processing. PV^+^ interneurons were selectively transfected with ChR2 using viral injections of the FLEXed ChR2 construct into auditory cortex of PV-Cre mice (Fig. 6*A*; see Materials and Methods). Blue light stimulation (150 ms, 473 nm, 5 mW) of cortical tissue caused transient activation of PV^+^ cells, which in turn produced strong inhibition of cortical pyramidal cells (Fig. 6*B*,*C*). This protocol allowed fast, temporally precise inactivation and recovery of auditory cortical processing. To confirm the fidelity of this approach, we first probed the effect of transiently inactivating cortex before the presentation of signals embedded in short duration maskers. When no early noise portion was present (“short”), we found that signal-evoked responses were unchanged following cortical disruption (Fig. 6*C*), with population signal responses demonstrating no effect of the laser manipulation (Fig. 6*C*,*D*). Overall, no significant difference was found between the mean change response functions, demonstrating that optogenetic perturbation had no effect on the signal response function (Friedman test, χ_(2)_^2^ = 3.73, *p* > 0.05). By contrast, laser-driven cortical inactivation during presentation of the early portion of the noise produced a visible reduction in signal evoked spiking responses in single cells (Fig. 7*A*). In addition, relatively large reductions in the population signal response were observed along with a reduction in sensitivity (Fig. 7*B*,*C*). This equated to a significant reduction in the signal response function (Friedman test, χ_(2)_^2^ = 6.65, *p* < 0.01). Laser stimulation during the presentation of the early sound portion also resulted in a large significant change in threshold, increasing thresholds by 5.4 dB (from 0.2 to 5.6 dB SNR; Fig. 7*D*). This increase in threshold was small but comparable in scale to the increases observed in short-CM maskers (Fig. 5). Selective optogenetic inactivation during preceding noise presentation caused a significant reduction in subsequent signal-evoked responses, demonstrating that auditory cortical processing is critical for large across-channel CMR in these neurons.

## Discussion

How the brain detects and segregates stimuli is an important question for understanding sensory perception. Here we have identified a neuronal correlate of across-channel CMR, an auditory phenomenon that improves signal detection, in A1 of the mouse. CM increases signal detectability in cortical neurons and also alters initial noise-evoked responses in a manner predictive of later masking release. Intriguingly, masking release is impaired by selective inactivation of A1 during early noise processing, suggesting that cortical processing is involved in the production of large across-channel CMR effects.

### Neuronal and behavioral correlates of across-channel CMR in animals

CMR has been demonstrated psychophysically in birds (Klump and Langemann, 1995; Langemann and Klump, 2001) and mammals (Kittel et al., 2000; Branstetter and Finneran, 2008; Klink et al., 2010; Trickey et al., 2010). Strong evidence for across-channel CMR has been found in the Starling (Langemann and Klump, 2001), but not in mouse (Klink et al., 2010). Klink et al. (2010) did observe that detection thresholds were lower in across-channel comodulated backgrounds, but these differences did not reach significance, in contrast to the relatively large neuronal CMR observed here. Differences in stimulus design may underlie this disparity: in the current study, we used maskers comprised of several (6) flanking noise bands (as opposed to 1) incorporating sinusoidal amplitude modulation (as opposed to low-pass noise). Such manipulations substantially increase the magnitude of CMR in humans (Buus, 1985; Hall et al., 1990; Buus et al., 1996; Verhey et al., 2003, ), suggesting, together with our neurophysiological data, that these acoustic features may be necessary to induce strong across-channel CMR in the mouse.

Our results provide evidence that across-channel processing contributes to forebrain correlates of CMR (Nelken et al., 1999; Las et al., 2005). However, as our recordings were performed under fentanyl-based anesthesia, it is not possible to know the extent to which our measurements represent perceptual and neuronal thresholds in behaving animals. The presence of a robust neuronal correlate of CMR under anesthesia does suggest that integration of temporal cues across frequency is a fundamental property of the ascending auditory pathway. However, top-down influences, such as general arousal (McGinley et al., 2015) and task-specific attention (Fritz et al., 2003), may modulate and further enhance signal detection in behaving animals.

### Modeling across-channel CMR

The most successful model of psychophysical across-channel CMR has been the equalization cancellation model (Buus, 1985; Piechowiak et al., 2007). This model uses ongoing temporal structure from one channel to cancel noise in the signal channel. In this scheme, cancellation of noise-evoked responses is predicted when comparing NB versus CM broadband conditions.

We find evidence of on-frequency noise suppression in the presence of flanking noise (NB to IM) and CM (IM to CM; Fig. 4*E*). However, our data demonstrate inconsistencies with the EC model. First, flanking noise should suppress ongoing responses to noise. Contrary to this, we found significant facilitation in ongoing responses to CM (and IM) maskers (Fig. 3), whereas suppression was only observed immediately following noise onset. Second, optogenetic manipulation disrupted A1 only during the early presentation period but preserved ongoing noise representation at the time of signal presentation. Ongoing cancellation processes as described in current EC models do not rely on stimulus history (with the exception of peripheral adaptation, which should be relatively unaffected by cortical disruption). Hence, suppressing cortical activity during the precursor should not affect ongoing cancellation. Ongoing cancellation has been previously observed in A1 (Fishman et al., 2012); however, we have used 10 Hz sinusoidal modulators (i.e., effectively 50 ms interstimulus intervals), which may induce stronger forward suppression (Wehr and Zador, 2005; Sadagopan and Wang, 2010) than stimuli with longer intervals. Our results indicate that active cancellation via ongoing lateral inhibition may be primarily a subcortical phenomenon (e.g., via wideband inhibition in cochlear nucleus) (Pressnitzer et al., 2001; Neuert et al., 2004; Ernst and Verhey, 2006). We also note that perceptual masking release can also occur in the absence of masking components at the signal frequency (Grzeschik et al., 2015), indicating that cancellation at signal frequency may not be the only way in which off-frequency cues contribute to CMR.

### Mechanisms underlying CMR in the brain

What processes underlie CMR in the brain? Studies in guinea pig cochlear nucleus and avian forebrain found that CM noise produces increased signal-evoked spiking activity (Pressnitzer et al., 2001; Hofer and Klump, 2003). In cat inferior colliculus, medial geniculate body, and A1, CM noise evokes decreases in noise-evoked activity at low SNRs, followed by, at much higher SNRs, increases in signal-evoked spiking (Nelken et al., 1999; Las et al., 2005). Our results concur with the former scheme, as CM overwhelmingly produced increases in signal-evoked spiking (although examples of decreases were observed). Previous studies also reported that most cortical cells demonstrate locking of spiking activity to the envelope of the noise (Nelken et al., 1999), which we have not observed. An explanation for this disparity is the spectral composition of the maskers used: Nelken et al. (1999) added spectral energy both within- and across-channel (whereas we added across-channel). Noise energy close to CF may result in enhanced noise locking. We suggest that within-channel CM noise produces more on-frequency locking than across-channel cues. If true, this could explain differences in the size of within- and across-channel CMR. Within-channel configurations produce behavioral CMR several times larger than across-channel CMR (Carlyon et al., 1989; Bacon et al., 1997; Verhey et al., 2003). Likewise, Nelken et al. (1999) observed locking suppression (decreased noise representation) at very low SNRs. With across-channel maskers, as used here, the probability of noise locking is reduced, which in turn reduces the opportunity for locking suppression. In a few cells demonstrating locking and responsive to the addition of a signal, we found examples of cells demonstrating locking suppression followed by increasing spiking to the signal, consistent with previous observations (Las et al., 2005). As locking suppression thresholds appear to be lower than signal thresholds, this would present a critical difference in the way in which the addition of a signal influences within- and across-channel configurations. Locking suppression may be more readily available in a within-channel configuration and hence lead to, on average, lower thresholds. In across-channel configurations, locking suppression would be available in fewer cells and lead to higher thresholds.

Consistent with psychophysical CMR, we observed that priming exposure to the masker improves signal representation in single A1 neurons. However, the mechanism(s) that underlie this effect remain unclear. In addition to producing strong masking release, CM maskers evoke weaker onset responses relative to NB and IM maskers. Cortical activity during the onset/early portion of the noise may be causally involved in the formation of CMR because transient and selective A1 inactivation strongly reduces masking release. However, the full expression of across channel CMR is not immediate (i.e., it takes time to build up). This latter observation suggests that slow adaptation to temporal coherence across-frequency is necessary to implement this form of CMR. The nature of such adaptation is not known but could include cortical synaptic depression (Carandini et al., 2002; Chung et al., 2002; Freeman et al., 2002), forward suppression (Wehr and Zador, 2005; Alves-Pinto et al., 2010; Scholes et al., 2011), and/or modulation at subcortical processing stations via cortical feedback (Bajo et al., 2007; Malmierca and Ryugo, 2011).

### Encoding of objects in the ascending auditory system

Overall we demonstrate that prior exposure to comodulated noise enhances signal detection. The auditory system is expert in quickly familiarizing itself with complex spectrotemporal patterns to allow discrimination/detection of changes (Elhilali and Shamma, 2008; McDermott and Simoncelli, 2011; Shamma et al., 2011; Krishnan et al., 2014; Andreou et al., 2015). A1 can quickly and specifically adapt to stereotyped spectrotemporal patterns while remaining nonadapted to similar, but nonstereotyped, patterns (Malone et al., 2015). This property may endow sensitivity to deviations in predictable sounds (Yaron et al., 2012; Kozlov and Gentner, 2014). Prior exposure of A1 to the spectrotemporal pattern of the masker is necessary for large across-channel CMR, supporting the idea that A1 might play a causal role more generally in the encoding of higher-order spectrotemporal features.

Neuronal encoding of sound becomes increasingly nonlinear throughout the ascending auditory system to the cortex, and evoked activity becomes more feature-selective. Abstraction processes may facilitate the extraction of features used to form auditory objects (Bizley and Cohen, 2013); and although these processes do not stop in A1 (Romanski et al., 2005; Sugihara et al., 2006; Teki et al., 2012), they are reasonably well developed at this stage (Bizley and Cohen, 2013; Mizrahi et al., 2014). Psychophysical studies of across-channel CMR demonstrate the dependence of this phenomenon upon features of object formation (McFadden and Wright, 1992; Grose and Hall, 1993; Dau et al., 2005, 2009; Verhey et al., 2012), and therefore suggest formation at higher centers of the ascending auditory system. Our results support this notion as specifically silencing auditory cortex during the presentation of crucial grouping cues (synchronous onset and early coherent modulation) has a large effect on the magnitude of across-channel CMR. This suggests that the formation of these cues could be occurring at the level of A1 or, cortical feedback during early stimulus exposure can facilitate this processing at subcortical locations.

Although our results support a role for auditory cortex, they also indicate that A1 is not solely responsible for the improvements in detectability afforded by across-channel CMR. This is implied by the observation that there is still a residual unmasking observed in our A1 inactivation condition (Figs. 5*D*, 7*D*). Pressnitzer et al. (2001) found cells in the cochlear nucleus demonstrating a correlate of across-channel CMR. This suggests that, like other abstract auditory features, cues associated with across-channel CMR are likely to be encoded along the ascending auditory system axis, but that cortical circuits may play a prominent role in their processing.

## References

[B1] Alves-Pinto A, Baudoux S, Palmer AR, Sumner CJ (2010). Forward masking estimated by signal detection theory analysis of neuronal responses in primary auditory cortex. J Assoc Res Otolaryngol.

[B2] Andreou LV, Griffiths TD, Chait M (2015). Sensitivity to the temporal structure of rapid sound sequences: an MEG study. Neuroimage.

[B3] Bacon SP, Lee J, Peterson DN, Rainey D (1997). Masking by modulated and unmodulated noise: effects of bandwidth, modulation rate, signal frequency, and masker level. J Acoust Soc Am.

[B4] Bajo VM, Nodal FR, Bizley JK, Moore DR, King AJ (2007). The ferret auditory cortex: descending projections to the inferior colliculus. Cereb Cortex.

[B5] Bathellier B, Ushakova L, Rumpel S (2012). Discrete neocortical dynamics predict behavioral categorization of sounds. Neuron.

[B6] Bizley JK, Cohen YE (2013). The what, where and how of auditory-object perception. Nat Rev Neurosci.

[B7] Branstetter BK, Finneran JJ (2008). Comodulation masking release in bottlenose dolphins (*Tursiops truncatus*). J Acoust Soc Am.

[B8] Buss E, Grose JH, Hall JW (2009). Features of across-frequency envelope coherence critical for comodulation masking release. J Acoust Soc Am.

[B9] Buus S (1985). Release from masking caused by envelope fluctuations. J Acoust Soc Am.

[B10] Buus S, Zhang L, Florentine M (1996). Stimulus-driven, time-varying weights for comodulation masking release. J Acoust Soc Am.

[B11] Buzsáki G, Mizuseki K (2014). The log-dynamic brain: how skewed distributions affect network operations. Nat Rev Neurosci.

[B12] Carandini M, Heeger DJ, Senn W (2002). A synaptic explanation of suppression in visual cortex. J Neurosci.

[B13] Carlyon RP, Buus S, Florentine M (1989). Comodulation masking release for three types of modulator as a function of modulation rate. J Acoust Soc Am.

[B14] Chung S, Li X, Nelson SB (2002). Short-term depression at thalamocortical synapses contributes to rapid adaptation of cortical sensory responses in vivo. Neuron.

[B15] Dau T, Ewert SD, Oxenham AJ (2005). Effects of concurrent and sequential streaming in comodulation masking release. Auditory signal processing.

[B16] Dau T, Ewert S, Oxenham AJ (2009). Auditory stream formation affects comodulation masking release retroactively. J Acoust Soc Am.

[B17] Elhilali M, Shamma SA (2008). A cocktail party with a cortical twist: how cortical mechanisms contribute to sound segregation. J Acoust Soc Am.

[B18] Ernst SM, Verhey JL (2006). Role of suppression and retro-cochlear processes in comodulation masking release. J Acoust Soc Am.

[B19] Fishman YI, Micheyl C, Steinschneider M (2012). Neural mechanisms of rhythmic masking release in monkey primary auditory cortex: implications for models of auditory scene analysis. J Neurophysiol.

[B20] Fletcher H (1940). Auditory patterns. Rev Modern Physics.

[B21] Freeman TC, Durand S, Kiper DC, Carandini M (2002). Suppression without inhibition in visual cortex. Neuron.

[B22] Fritz J, Shamma S, Elhilali M, Klein D (2003). Rapid task-related plasticity of spectrotemporal receptive fields in primary auditory cortex. Nat Neurosci.

[B23] Grose JH, Hall JW (1993). Comodulation masking release: is comodulation sufficient?. J Acoust Soc Am.

[B24] Grose JH, Hall JW, J, Buss E (2005a). Across-channel spectral processing. Int Rev Neurobiol.

[B25] Grose JH, Hall JW, Buss E, Hatch DR (2005b). Detection of spectrally complex signals in comodulated maskers: effect of temporal fringe. J Acoust Soc Am.

[B26] Grose JH, Buss E, Hall JW (2009). Within-and across-channel factors in the multiband comodulation masking release paradigm. J Acoust Soc Am.

[B27] Grzeschik R, Lübken B, Verhey JL (2015). Comodulation masking release in an off-frequency masking paradigm. J Acoust Soc Am.

[B28] Hall JW, Haggard MP, Fernandes MA (1984). Detection in noise by spectro-temporal pattern analysis. J Acoust Soc Am.

[B29] Hall JW, Grose JH, Haggard MP (1990). Effects of flanking band proximity, number, and modulation pattern on comodulation masking release. J Acoust Soc Am.

[B30] Harris KD, Bartho P, Chadderton P, Curto C, de la Rocha J, Hollender L, Itskov V, Luczak A, Marguet SL, Renart A, Sakata S (2011). How do neurons work together? Lessons from auditory cortex. Hear Res.

[B31] Hatch DR, Arné BC, Hall JW (1995). Comodulation masking release (CMR): effects of gating as a function of number of flanking bands and masker bandwidth. J Acoust Soc Am.

[B32] Hofer SB, Klump GM (2003). Within-and across-channel processing in auditory masking: a physiological study in the songbird forebrain. J Neurosci.

[B33] Kittel M, Wagner E, Klump G (2000). Hearing in the gerbil (*Meriones unguiculatus*): comodulation masking release. Zoology.

[B34] Klink KB, Dierker H, Beutelmann R, Klump GM (2010). Comodulation masking release determined in the mouse (*Mus musculus*) using a flanking-band paradigm. J Assoc Res Otolaryngol.

[B35] Klump GM, Langemann U (1995). Comodulation masking release in a songbird. Hear Res.

[B36] Klumpp R, Eady H (1956). Some measurements of interaural time difference thresholds. J Acoust Soc Am.

[B37] Kozlov AS, Gentner TQ (2014). Central auditory neurons display flexible feature recombination functions. J Neurophysiol.

[B38] Krishnan L, Elhilali M, Shamma S (2014). Segregating complex sound sources through temporal coherence. PLoS Comput Biol.

[B39] Langemann U, Klump GM (2001). Signal detection in amplitude-modulated maskers: I. Behavioural auditory thresholds in a songbird. Eur J Neurosci.

[B40] Las L, Stern EA, Nelken I (2005). Representation of tone in fluctuating maskers in the ascending auditory system. J Neurosci.

[B41] Linden JF, Liu RC, Sahani M, Schreiner CE, Merzenich MM (2003). Spectrotemporal structure of receptive fields in areas AI and AAF of mouse auditory cortex. J Neurophysiol.

[B42] Malmierca MS, Ryugo DK (2011). Descending connections of auditory cortex to the midbrain and brain stem. The auditory cortex.

[B43] Malone BJ, Beitel RE, Vollmer M, Heiser MA, Schreiner CE (2015). Modulation-frequency-specific adaptation in awake auditory cortex. J Neurosci.

[B44] May BJ, Kimar S, Prosen CA (2006). Auditory filter shapes of CBA/CaJ mice: behavioral assessments. J Acoust Soc Am.

[B45] McDermott JH, Simoncelli EP (2011). Sound texture perception via statistics of the auditory periphery: evidence from sound synthesis. Neuron.

[B46] McDermott JH, Schemitsch M, Simoncelli EP (2013). Summary statistics in auditory perception. Nat Neurosci.

[B47] McFadden D, Wright BA (1990). Temporal decline of masking and comodulation detection differences. J Acoust Soc Am.

[B48] McFadden D, Wright BA (1992). Temporal decline of masking and comodulation masking release. J Acoust Soc Am.

[B49] McGinley MJ, David SV, McCormick DA (2015). Cortical membrane potential signature of optimal states for sensory signal detection. Neuron.

[B50] Mizrahi A, Shalev A, Nelken I (2014). Single neuron and population coding of natural sounds in auditory cortex. Curr Opin Neurobiol.

[B51] Nelken I (2014). Stimulus-specific adaptation and deviance detection in the auditory system: experiments and models. Biol Cybern.

[B52] Nelken I, Rotman Y, Bar Yosef O (1999). Responses of auditory-cortex neurons to structural features of natural sounds. Nature.

[B53] Neuert V, Verhey JL, Winter IM (2004). Responses of dorsal cochlear nucleus neurons to signals in the presence of modulated maskers. J Neurosci.

[B54] Nieder A, Klump GM (2001). Signal detection in amplitude-modulated maskers: II. Processing in the songbird's auditory forebrain. Eur J Neurosci.

[B55] Piechowiak T, Ewert SD, Dau T (2007). Modeling comodulation masking release using an equalization-cancellation mechanism. J Acoust Soc Am.

[B56] Plomp R (1964). Rate of decay of auditory sensation. J Acoust Soc Am.

[B57] Pressnitzer D, Meddis R, Delahaye R, Winter IM (2001). Physiological correlates of comodulation masking release in the mammalian ventral cochlear nucleus. J Neurosci.

[B58] Romanski LM, Averbeck BB, Diltz M (2005). Neural representation of vocalizations in the primate ventrolateral prefrontal cortex. J Neurophysiol.

[B59] Rosenblith WA, Stevens KN (1953). On the DL for frequency. J Acoust Soc Am.

[B60] Sadagopan S, Wang X (2010). Contribution of inhibition to stimulus selectivity in primary auditory cortex of awake primates. J Neurosci.

[B61] Scholes C, Palmer AR, Sumner CJ (2011). Forward suppression in the auditory cortex is frequency-specific. Eur J Neurosci.

[B62] Shamma SA, Elhilali M, Micheyl C (2011). Temporal coherence and attention in auditory scene analysis. Trends Neurosci.

[B63] Sugihara T, Diltz MD, Averbeck BB, Romanski LM (2006). Integration of auditory and visual communication information in the primate ventrolateral prefrontal cortex. J Neurosci.

[B64] Sutter ML, Schreiner CE (1991). Physiology and topography of neurons with multipeaked tuning curves in cat primary auditory cortex. J Neurophysiol.

[B65] Taaseh N, Yaron A, Nelken I (2011). Stimulus-specific adaptation and deviance detection in the rat auditory cortex. PLoS One.

[B66] Teki S, Kumar S, von Kriegstein K, Stewart L, Lyness CR, Moore BC, Capleton B, Griffiths TD (2012). Navigating the auditory scene: an expert role for the hippocampus. J Neurosci.

[B67] Teki S, Chait M, Kumar S, Shamma S, Griffiths TD (2013). Segregation of complex acoustic scenes based on temporal coherence. Elife.

[B68] Trickey JS, Branstetter BK, Finneran JJ (2010). Auditory masking of a 10 kHz tone with environmental, comodulated, and Gaussian noise in bottlenose dolphins (*Tursiops truncatus*). J Acoust Soc Am.

[B69] Ulanovsky N, Las L, Nelken I (2003). Processing of low-probability sounds by cortical neurons. Nat Neurosci.

[B70] Verhey JL, Pressnitzer D, Winter IM (2003). The psychophysics and physiology of comodulation masking release. Exp Brain Res.

[B71] Verhey JL, Ernst SM, Yasin I (2012). Effects of sequential streaming on auditory masking using psychoacoustics and auditory evoked potentials. Hear Res.

[B72] Wehr M, Zador AM (2005). Synaptic mechanisms of forward suppression in rat auditory cortex. Neuron.

[B73] Yaron A, Hershenhoren I, Nelken I (2012). Sensitivity to complex statistical regularities in rat auditory cortex. Neuron.

